# A Case of Severe Generalized Granuloma Annulare Responding Well to Tofacitinib

**DOI:** 10.1155/crdm/3593724

**Published:** 2025-05-10

**Authors:** Saman Al-Zahawi, Narges Ghandi, Vahidesadat Azhari, Kamran Balighi

**Affiliations:** ^1^Department of Dermatology, Razi Hospital, Tehran University of Medical Sciences (TUMS), Tehran, Iran; ^2^Autoimmune Bullous Diseases Research Center, Razi Hospital, Tehran University of Medical Sciences, Tehran, Iran; ^3^Department of Dermatopathology, Razi Hospital, Tehran University of Medical Sciences (TUMS), Tehran, Iran

**Keywords:** drug therapy, GA, granuloma annulare, hepatitis B, Janus kinase inhibitors, tofacitinib

## Abstract

Granuloma annulare is a noninfectious, granulomatous skin disorder that typically presents as symmetrical, asymptomatic, annular, or arciform plaques with slightly raised edges, often found on the back of the hands or feet. This report highlights the successful use of tofacitinib in treating resistant/generalized granuloma annulare in a 66-year-old male with a known history of hepatitis B, demonstrating its potential as an effective therapeutic option.

## 1. Introduction

Granuloma annulare (GA) is a noninfectious, granulomatous skin condition that typically presents as symmetrical, asymptomatic, annular, or arciform plaques with slightly raised borders, commonly found on the dorsum of the hands or feet. These plaques may be localized to the hands and feet or appear in a generalized form, involving the trunk, extremities, and, in rare cases, the face. Less common clinical variants include subcutaneous GA, which primarily occurs in children and can resemble rheumatoid nodules [1]; macular GA, which typically involves symmetrical areas of the proximal extremities [2]; atypical GA, which can appear in unusual locations such as the palms or soles and may be associated with underlying malignancy or HIV infection [3]; papular GA; and perforating GA, which may be mistaken for perforating collagen disorders. According to the literature, more than two-thirds of GA cases occur in individuals younger than 30 years [4]. However, recent studies suggest that GA is more commonly seen in individuals in their fourth to fifth decade of life, with a female-to-male ratio of 3:1 [5].

Histologically, GA is characterized by infiltrating or palisading histiocytes in the superficial or deep dermis, surrounding areas of collagen breakdown. The presence of dermal mucin deposition serves as a key diagnostic feature in histological examination [6].

The treatment approach for GA typically begins with intralesional corticosteroids as the first-line therapy for localized cases. For more generalized or extensive presentations, the treatment escalates to systemic medications, such as hydroxychloroquine (HCQ), or phototherapy.

## 2. Case Report

**Figure 1:**
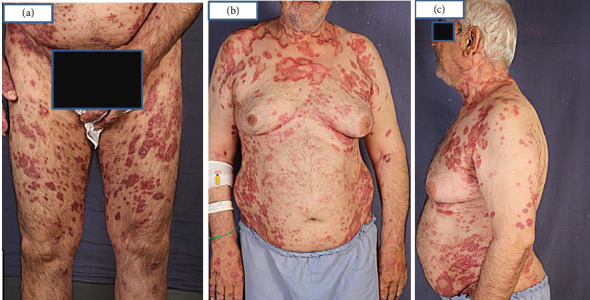
At admission, erythematous annular plaques with central clearance coalescing together in the lower extremity (a), upper trunk lesions resemble SCLE (b), and involvement of neck and face (c), which is rare in GA.

**Figure 2:**
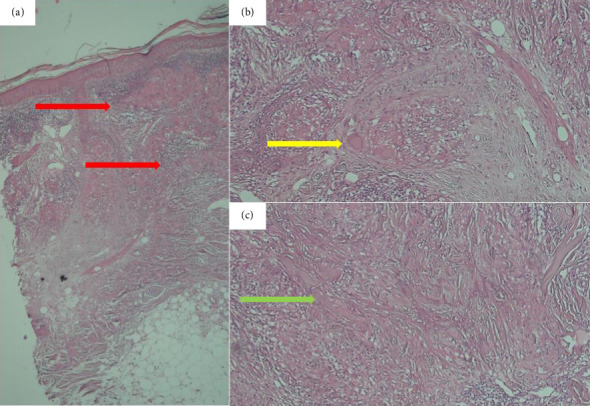
Histological examination of a trunk punch biopsy, stained with hematoxylin and eosin (H&E), revealed an infiltration of the superficial and deep dermis by epithelioid histiocytes (red arrow) at 100x magnification (a). At a higher magnification (200x, (b)), multinucleated giant cells, including both Langhans and foreign body types (yellow arrow), were observed alongside lymphocytes. Additionally, patchy areas of collagen breakdown (collagenolysis) were evident (green arrow, (c)).

A 66-year-old male, a heavy smoker with a history of addiction, presented with a widespread skin eruption affecting the face, trunk, and both upper and lower extremities (Figures 1(a), 1(b), and 1(c)). Two years prior, he was diagnosed with GA based on clinical appearance and biopsy findings. At that time, treatment with topical clobetasol and intralesional corticosteroids failed to control the skin eruption. Due to the progression of the lesions, lack of response to topical corticosteroids, and the need to exclude other dermatological conditions, a second biopsy was performed. The punch biopsy from the trunk lesions confirmed the diagnosis of GA (Figures 2(a), 2(b), and 2(c)). The patient was subsequently treated with HCQ for 1 year, but the lesions continued to expand without improvement. Ultimately, he was admitted to our dermatology center for severe, generalized skin eruption involving the entire body.

Upon examination, generalized, annular, and arciform erythematous plaques were observed on the trunk, extremities, and face. No mucosal lesions were noted.

Laboratory tests, including complete blood count, liver function test, renal function test, LDH, thyroid function test, and electrolyte levels, showed no abnormalities at the time of admission. Virology testing for hepatitis revealed a positive Hbc Ab result, and the patient was prescribed tenofovir 300 mg daily. Given the potential association between GA and hepatitis B, the patient was maintained solely on antiviral therapy for 3 months to monitor whether the skin eruption would resolve. However, the skin lesions remained persistent and unchanged throughout the initial 3 months of tenofovir treatment. The widespread and severe nature of the skin lesions, combined with the patient's reluctance to undergo phototherapy and the lack of response to HCQ and other conventional treatments, led us to consider JAK inhibitors as a therapeutic option. This decision was made while carefully considering the patient's positive hepatitis status.

**Figure 3:**
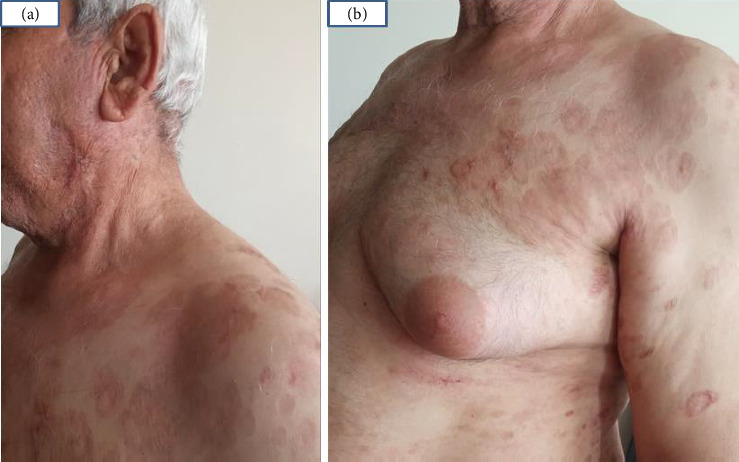
Noticeable clearing of the plaques on the neck, trunk, and face was observed (a and b), with only residual hyperpigmentation remaining at the sites of the former lesions.

The decision to use a JAK inhibitor was based on recent studies highlighting the role of the Janus kinase-signal transducer and activator of transcription (JAK-STAT) pathway in the pathogenesis of GA [6]. Tofacitinib 5 mg twice daily was initiated after completing initial laboratory tests and ruling out potential contraindications for JAK inhibitors. Within 1 month of starting the treatment, a significant improvement was observed, with complete resolution of all lesions, leaving only postinflammatory hyperpigmentation (Figures 3(a) and 3(b)).

This case report describes the remarkable response of resistant generalized GA in a 66-year-old male with hepatitis B, showing near-complete clearance of lesions just one month after beginning tofacitinib therapy.

## 3. Discussion

Generalized GA is a rare subtype of GA, typically presenting later in life compared to other forms of GA and often showing a poorer response to conventional treatments. While its exact cause remains unknown, generalized GA has been linked to various factors, including pneumococcal and COVID-19 vaccination [7, 8], dyslipidemia, diabetes mellitus, malignancy, thyroid disease, and hepatitis C and B [9]. In this case, hepatitis B may have been associated with the generalized GA, but treating the underlying condition for 3 months did not improve the skin lesions. Dyslipidemia and diabetes mellitus were ruled out based on normal lipid profiles and blood sugar levels. Certain medications, aside from vaccines, have also been associated with generalized GA. For instance, both the induction and successful treatment of generalized GA with dupilumab have been reported [10, 11]. Additionally, generalized GA has been documented as a paraneoplastic sign of Hodgkin lymphoma relapse [12].

Clinically, diagnosing classic GA plaques is straightforward. However, the widespread distribution of lesions in this case, particularly involving the upper trunk, necessitated histopathological evaluation to rule out subacute cutaneous lupus erythematosus (SCLE) and other similar dermatoses. Pathological findings revealed granulomatous changes characteristic of GA. These findings, coupled with the absence of vacuolar changes, effectively ruled out SCLE. Although other granulomatous conditions, such as sarcoidosis and necrobiosis lipoidica, can exhibit similar changes, the presence of mucin in approximately 90% of GA cases aids in distinguishing it. Furthermore, sarcoidosis is characterized by minimal lymphocytic infiltration and “naked” granulomas. Lastly, necrobiosis lipoidica, which presents with epidermal atrophy, vasculopathy, and lipid deposition, was excluded due to the absence of these features.

Untreated localized GA may resolve spontaneously within 2 years, though recurrence, particularly at the original site, is common. For patients seeking treatment, factors such as whether the GA is localized or generalized, associations with other diseases, and medication history should be considered. Asymptomatic localized lesions may only require monitoring. For symptomatic or bothersome localized lesions, intralesional corticosteroids are the first-line treatment. Other topical therapies with less evidence of efficacy include corticosteroids, calcineurin inhibitors, and imiquimod. Generalized GA, however, often requires more advanced approaches. Although generalized GA tends to respond poorly to treatment compared to localized GA, HCQ and PUVA therapy have shown effectiveness. In this case, the patient received HCQ 200 mg twice daily for nearly a year without improvement and experienced further lesion expansion. PUVA, a reliable option for generalized GA, was impractical for this patient due to his inability to attend regular phototherapy sessions [13]. Methotrexate, while capable of causing generalized papular eruptions with granulomatous changes in patients with rheumatoid arthritis [14], has also been reported to successfully treat generalized GA in previous studies [15].

The rationale for utilizing a JAK inhibitor was grounded in recent research that identified the JAK-STAT pathway as a key player in the pathogenesis of GA [6]. This choice is further supported by the mechanism of action of JAK inhibitors. Unlike corticosteroids and HCQ, which exert effects on a limited number of cytokines, JAK inhibitors possess the ability to simultaneously block multiple cytokines. This broader cytokine inhibition likely contributes to their observed efficacy in our study. Furthermore, a study of 15 patients with generalized GA treated with tofacitinib (5 mg twice daily), including 9 with prior conventional therapy, demonstrated that tofacitinib was effective, achieving complete clearance in 11 patients (mean time: 4.4 months) and partial clearance in 4 patients. The authors concluded that tofacitinib is a beneficial JAK-STAT inhibitor for GA, especially in generalized and recalcitrant cases [16].

Previous studies have also documented the efficacy of baricitinib and upadacitinib in resolving generalized GA [17, 18]. Other recent treatments for GGA include apremilast, excimer laser, and alitretinoin [19–21]. Additionally, the resolution of generalized GA following biopsy, known as the remote reverse Koebner phenomenon, has been reported [22]. Although this patient showed a remarkable response to treatment, several challenges remain, including determining the optimal duration of therapy, the timing for tapering, and the risk of GA recurrence after discontinuing JAK inhibitors. These aspects require further research and long-term follow-up to better understand their implications.

## 4. Conclusion

Generalized GA tends to have a more prolonged course and a lower response to treatment compared to other subtypes of GA. JAK inhibitors may be used as an alternative systemic medication in resistant generalized GA.

## Data Availability

The data that support the findings of this study are available on request from the corresponding author. The data are not publicly available due to privacy or ethical restrictions.
